# Supplementary figures and images for: Perturbations of the ZED1 pseudokinase activate plant immunity
Source: PLoS Pathog. 2019 Jul 3;15(7):e1007900. doi: 10.1371/journal.ppat.1007900 (PMC6634424; doi:10.1371/journal.ppat.1007900)

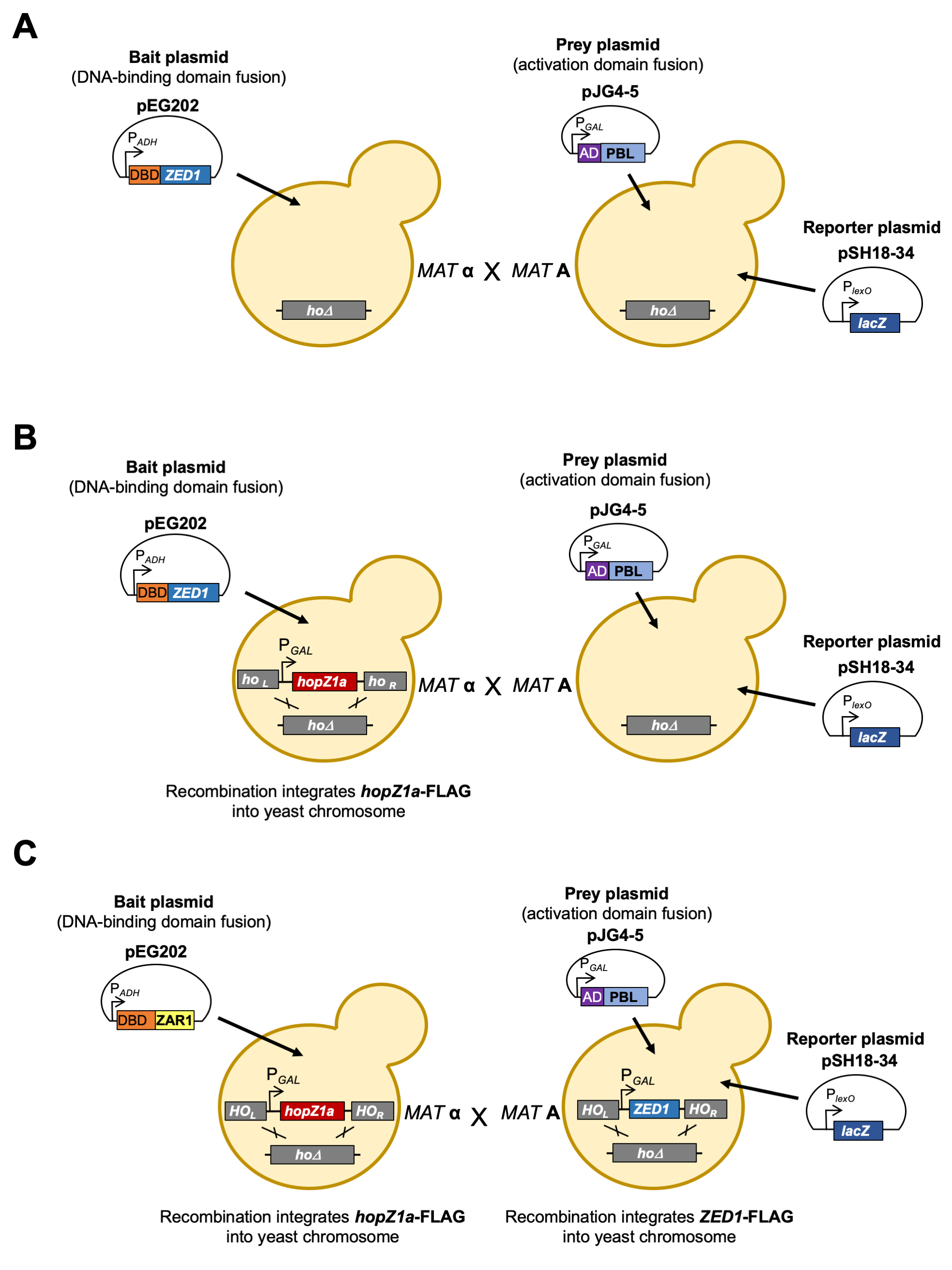

Supplement: S1 Fig — (A) Y2H: Transformants of haploid yeast strain EGY48 (MAT α) carrying pEG202 bait fusion plasmids (in this example, pEG202::lexADBD-ZED1) are mated with transformants of haploid yeast strain RFY206 (MAT A) carrying pJG4-5 prey fusion plasmids (pJG4-5::AD-PBL; AD = NLS-B42AD-HA) and the reporter plasmid pSH18-34 (lacZ). (B) Y3H: Genes of interest (in this example hopZ1a) are integrated at the ho locus of the haploid yeast strain EGY48 (MAT α; see Materials and Methods). These strains were subsequently transformed with pEG202 bait fusion plasmids (in this example, pEG202::lexADBD-ZED1) and mated with transformants of haploid yeast strain RFY206 (MAT A) carrying prey fusion plasmids (pJG4-5::AD-PBL) and the reporter plasmid pSH18-34. (C) Y4H: Transformants of haploid yeast strain EGY48 (MAT α) with integrated hopZ1a alleles and carrying pEG202 bait fusion plasmids (pEG202::lexADBD-ZAR1) are mated with transformants of haploid yeast strain RFY206 (MAT A) with integrated ZED1wt and carrying prey fusion plasmids (pJG4-5::AD-PBL) and the reporter plasmid pSH18-34. (TIF) [file ppat.1007900.s001.tif]

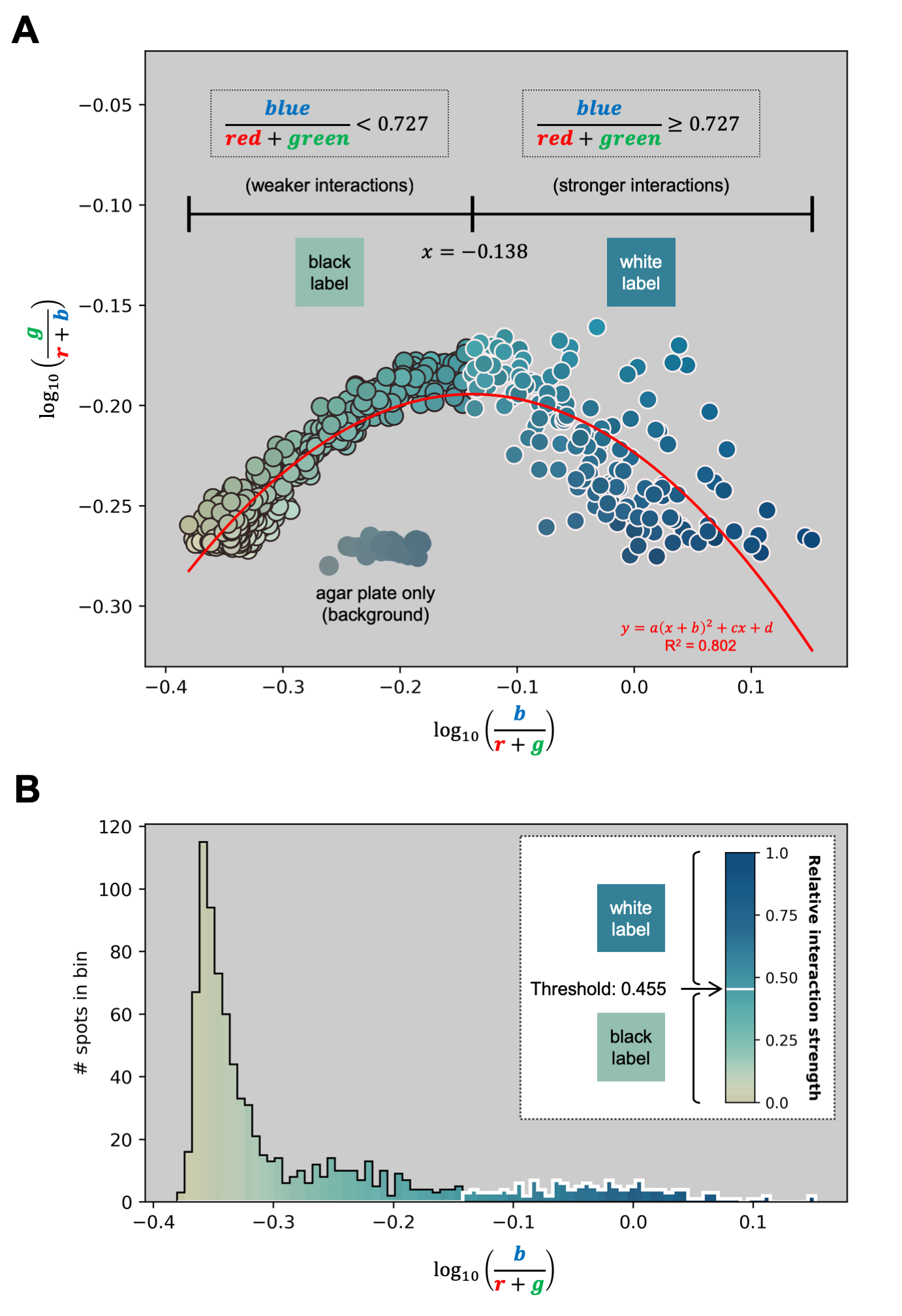

Supplement: S2 Fig — (A) Averaged RGB pixel values for each of the yeast colonies presented in Fig 2, S4 Fig, S5 Fig, S8 Fig and S9 Fig (see Materials and Methods) are plotted as a function of the log-transformed relative intensities of their blue (x-axis) and green (y-axis) channels. This plotting strategy results in a smooth arc describing the transition between strong (mostly blue) and weak (yellowish white) interactions, while spots representing array positions with no yeast colonies (agar plate only) form a distinct cluster below. The data were fitted to a quadratic function of the form y = a(x+b)2+cx+d (plotted as a red line; a = −1.511, b = 0.67, c = 1.607, d = 0.456; R2 = 0.802), and the point at which this curve reaches its maximum (x=−0.138;bluered+green=0.727) was used a threshold for discriminating strong interactions from bait-prey pairs with only weak-to-moderate interaction strength. Spots exceeding this threshold are shown with a white border and are assigned white labels in the array layouts accompanying Fig 1A, Fig 2, Fig 5, S4 Fig, S5 Fig, S8 Fig, S9 Fig, and S11 Fig. (B) Histogram of the x-coordinates of all yeast spots plotted in panel A (excluding agar-only background spots). A consensus colour for the histogram bars representing each of 85 bins was determined by finding the average red, green, and blue channel intensities for all of the spots represented by a given bin. Bins exceeding the threshold described in panel A are highlighted with a white border. Inset—a colour-bar derived from the histogram data relating averaged pixel colours to a ‘relative interaction strength’ metric; the log-transformed relative intensities of the blue channel for each spot (−0.38≤x≤0.15) were scaled to values between 0 and 1. Empty bins (bins 76, 80, 81, 82, 83, and 84) were assigned the colour of the immediately-preceding non-zero bin, and the resulting colour range was smoothed by applying a five-bin sliding window average. The value corresponding to the white/black label threshol [file ppat.1007900.s002.tif]

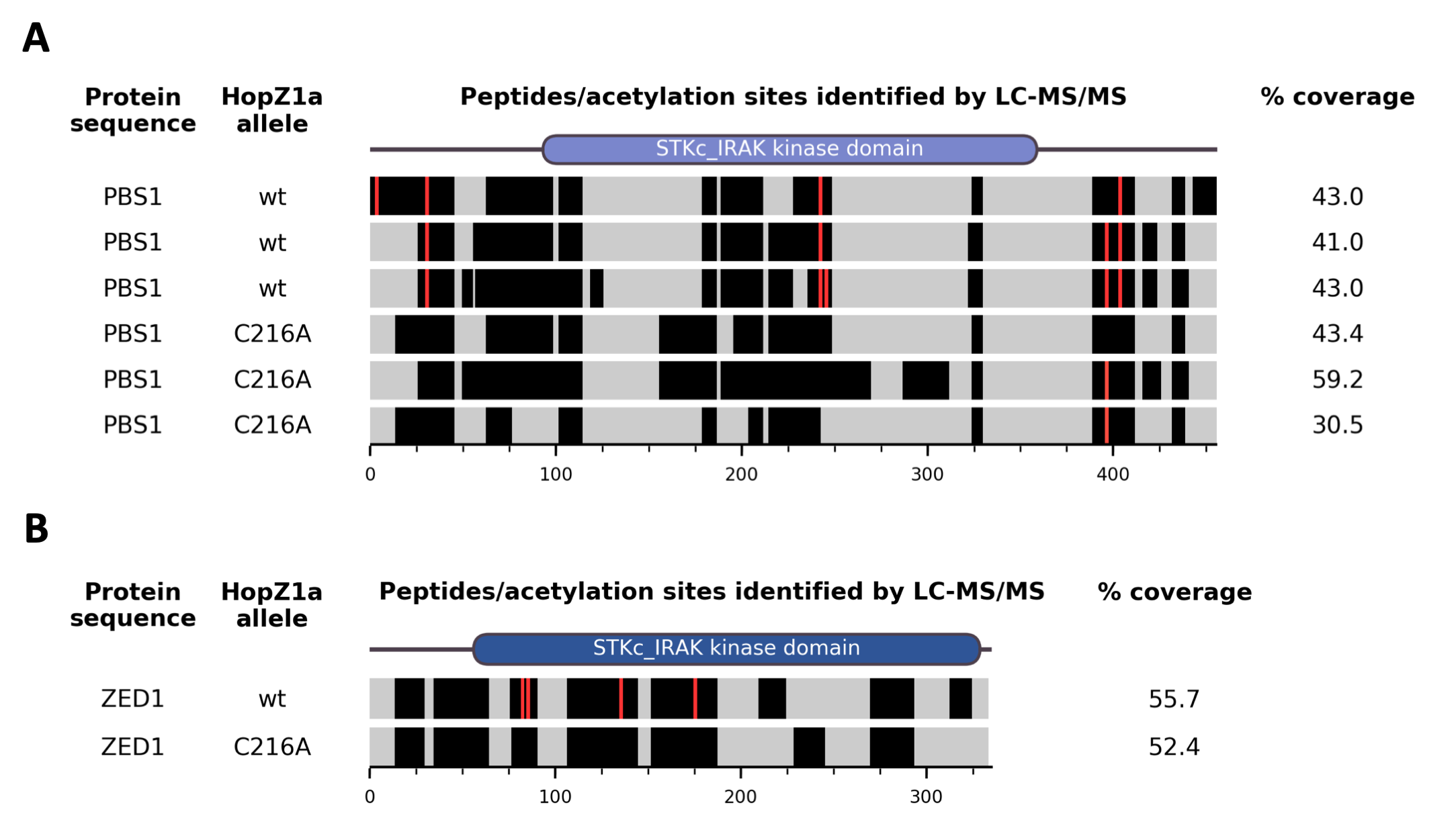

Supplement: S3 Fig — Peptides and acetylation sites from PBS1 (A) and ZED1 (B) are plotted as in Fig 1B and 1C, except for the additional inclusion of peptides that only surpass the ‘Low’ confidence threshold reported by the Proteome DiscovererTM software, as described in S1 File. Note that the acetylation of PBS1 S398 suggested by two of the samples from co-expression with HopZ1aC216A may be due to the activity of endogenous yeast acetyltransferases, since previous reports by ourselves [92] and others [93–95] indicate that this mutant is devoid of both trans-acetylation and autoacetylation activities, as assessed by gel-based in vitro acetylation assays with purified recombinant proteins. (TIF) [file ppat.1007900.s003.tif]

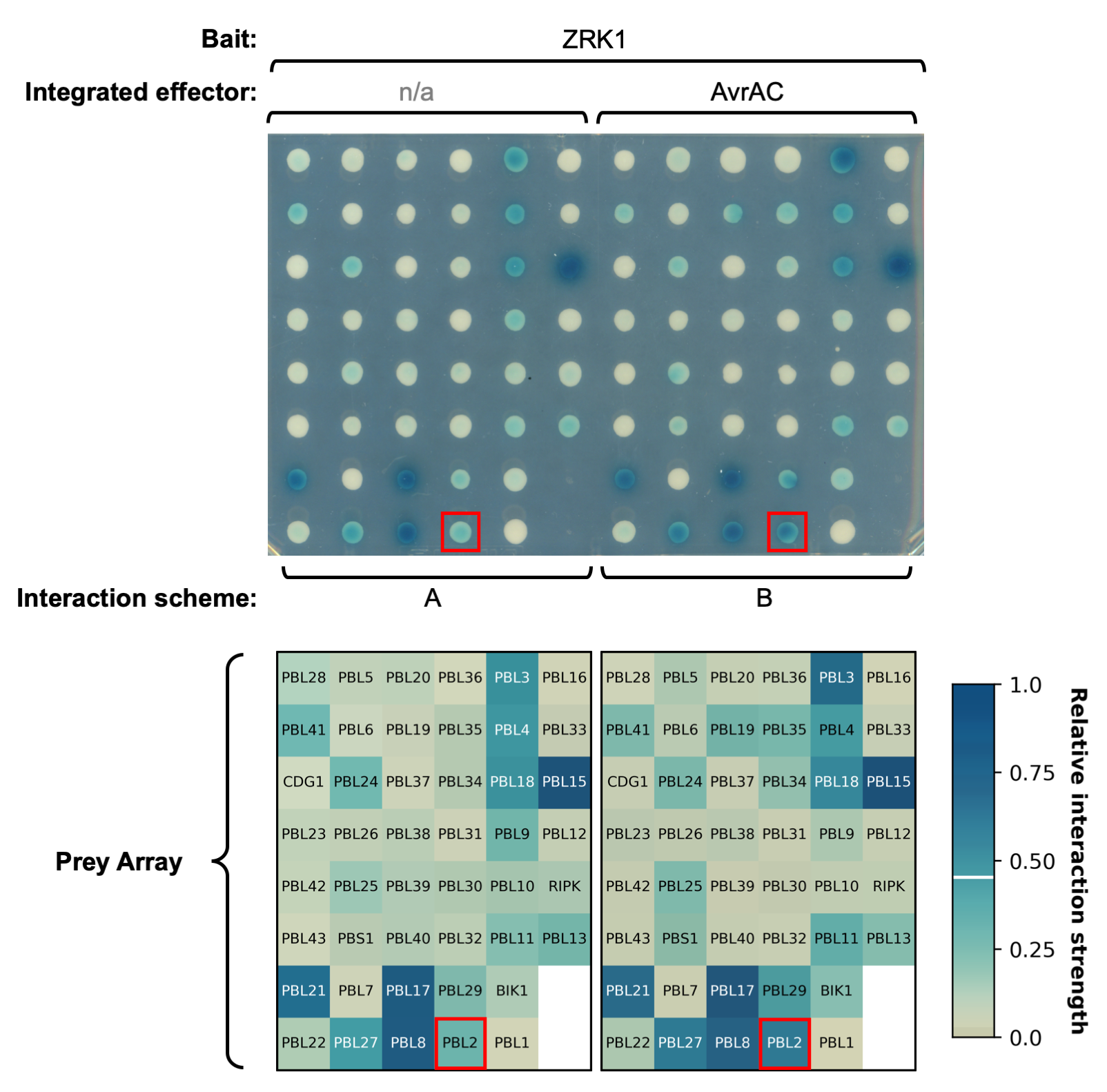

Supplement: S4 Fig — Top—Binding of ZRK1/RKS1 to 46 PBLs was assessed in the absence (Y2H; left) or presence (Y3H; right) of the X. campestris effector AvrAC; interaction schemes refer to S1 Fig. Bottom–Prey array layouts showing the relative strength of interactions between ZRK1/RKS1 and PBLs, corresponding to the X-gal reporter plate above. Label colours (white or black) for each array position are determined by the relative interaction strength (see S2 Fig; Materials and Methods). Yeast spots and array positions corresponding to the positive control interaction between ZRK1/RKS1 and PBL2 are highlighted with a red frame. (TIF) [file ppat.1007900.s004.tif]

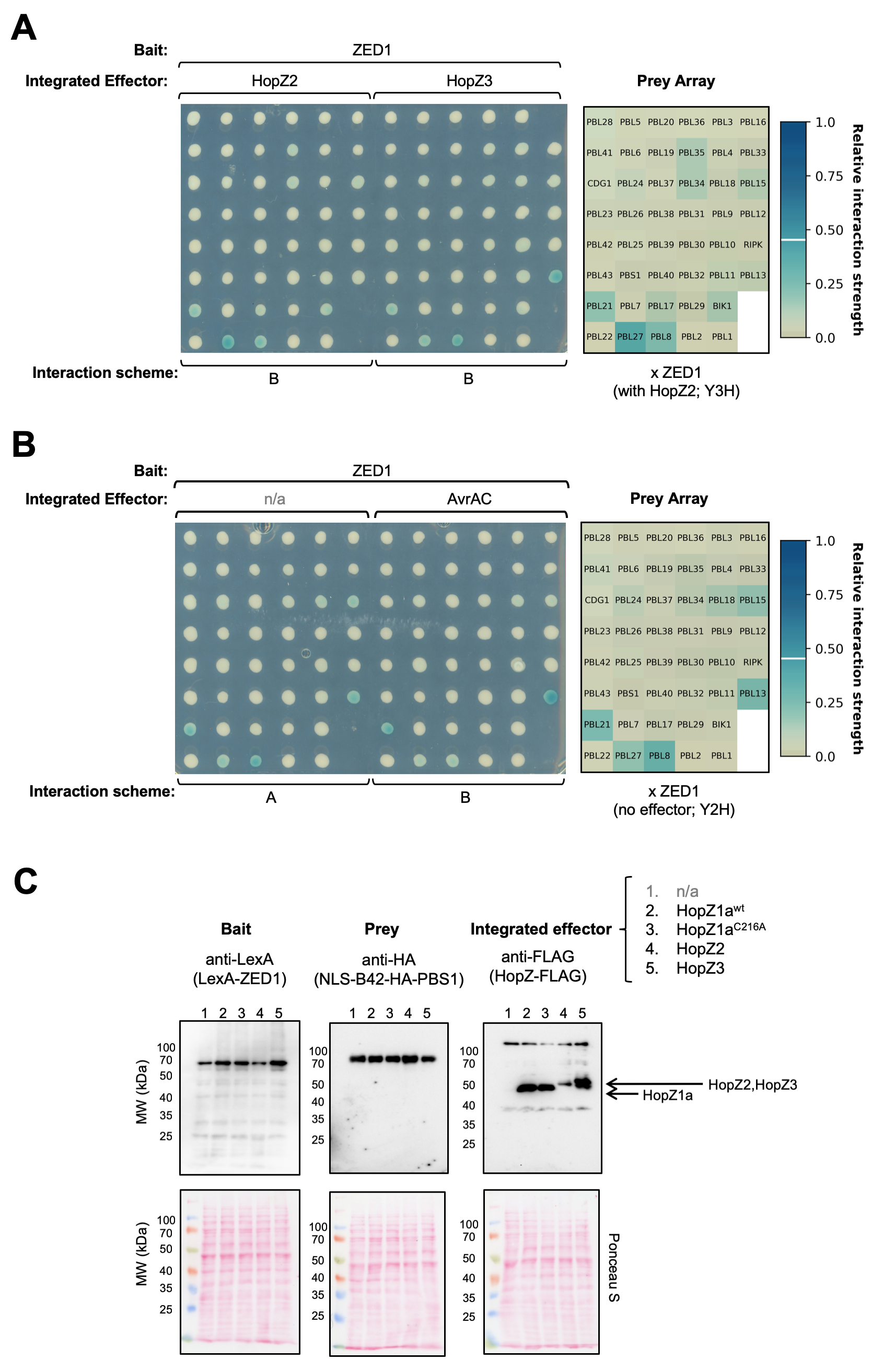

Supplement: S5 Fig — Y2H interactions between ZED1 and PBLs in the absence of effectors (panel B, left) are compared with Y3H interactions in the presence of the indicated effectors (panel A; panel B, middle). Prey array layouts at right indicate the relative strength of ZED-PBL interactions in the presence of HopZ2 (A) and in the absence of effector (B). All array labels are black since the relative interaction strengths are all below the threshold described in S2 Fig. Interaction schemes refer to S1 Fig. (C) Top—Representative western blots showing consistent expression of both bait (ZED1), prey (PBS1), and integrated effectors (HopZ alleles). Bottom–Nitrocellulose membranes stained with Ponceau S demonstrate equal loading of yeast cell extracts and consistent transfer from SDS-PAGE gels across all lanes. (TIF) [file ppat.1007900.s005.tif]

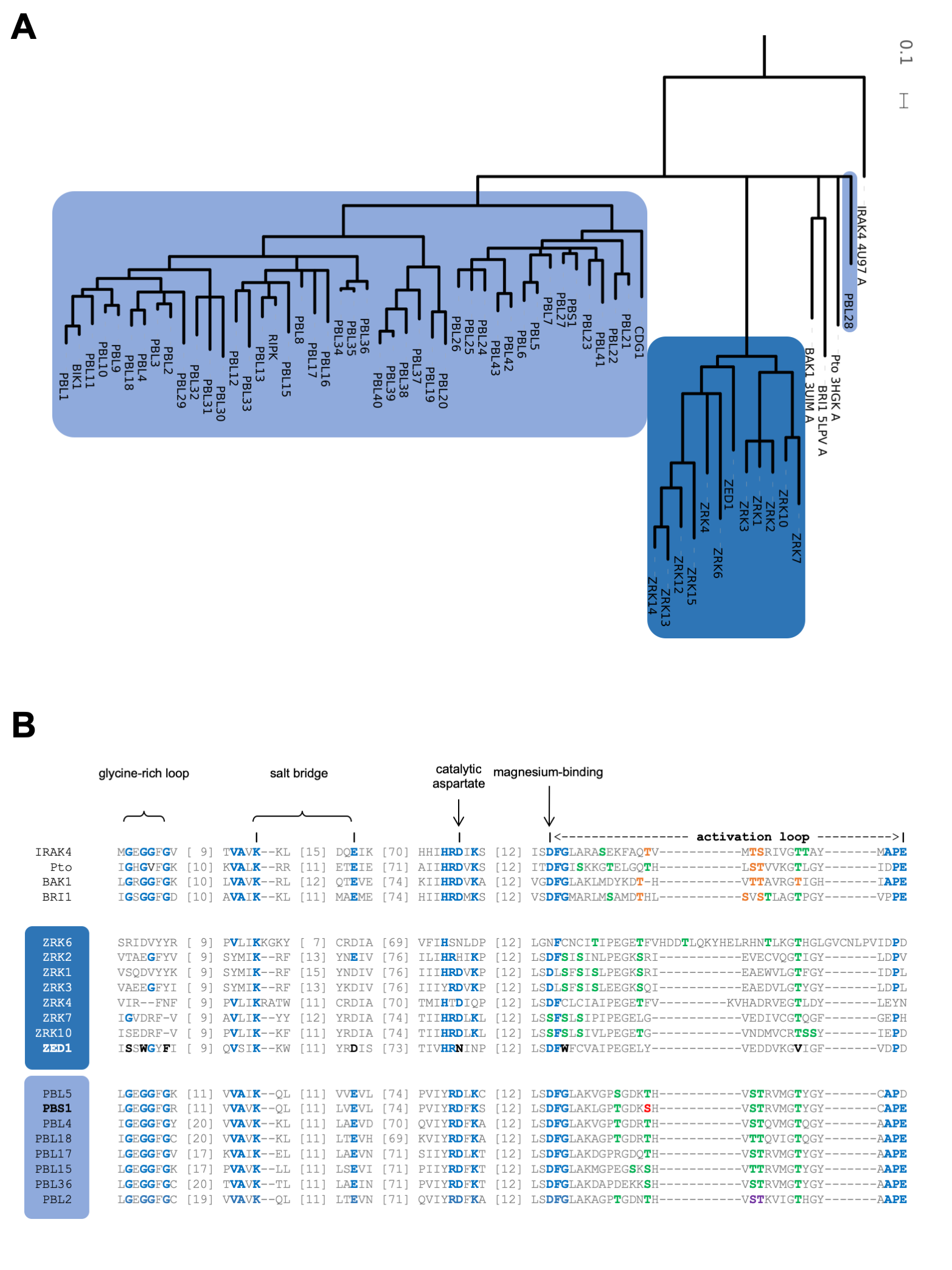

Supplement: S6 Fig — (A) Phylogenetic tree showing evolutionary relationships between the kinase domains of ZED1/ZRKs (dark blue shading), PBLs (light blue shading), and sequences from four structurally-characterized kinase domains—Arabidopsis BRI1 (PDB: 5LPV) [35] and BAK1 (PDB: 3UIM) [36], Solanum pimpinellifolium (tomato) Pto kinase (PDB: 3HGK) [76], and human IRAK4 (PDB: 4U97) [44]. Note that PBL28 is an out-group compared to other PBLs and may therefore not represent a ‘true’ PBL. (B) A subset of columns from the alignment used to generate the tree shown in panel A are presented to highlight important kinase motifs present in structurally-characterized kinase domains (top), ZED1/ZRKs (middle), and a representative subset of PBLs (bottom). The numbers of omitted, non-gap positions between adjacent blocks of consecutive columns are shown between square brackets for each sequence. Highlighted in blue are exact matches to established kinase motifs. Serine and threonine residues between the ‘DFG’ and ‘APE’ motifs that define the activation loop are highlighted in green. Serine/threonine residues that have been observed in phosphorylated form in previously-described crystal structures are highlighted in orange. PBS1 S244, a site acetylated by HopZ1a, and immediately following the site cleaved by HopAR1, is highlighted in red. PBL2 S253 and T254, sites uridylated by the X. campestris effector AvrAC and required for AvrAC-induced interaction with ZRK1/RKS1 [14], are highlighted in purple. ZED1 residues targeted for mutagenesis in this study are indicated in bold, black font. (TIF) [file ppat.1007900.s006.tif]

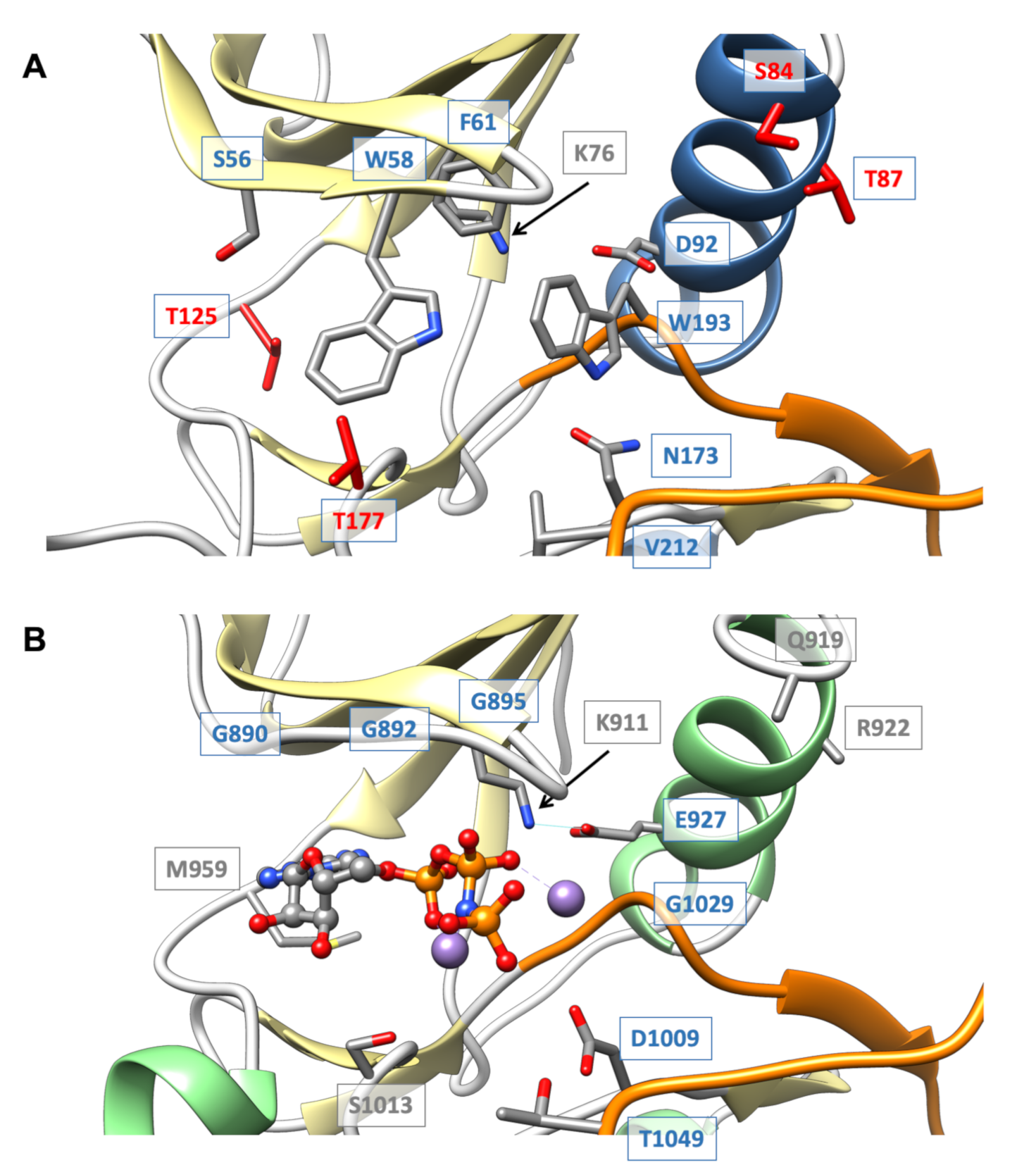

Supplement: S7 Fig — (A) Homology-based structural model (see Materials and Methods) of the ZED1 active site showing the relative positions of the sites targeted for mutagenesis in this study. Blue residue labels indicate positions that were mutated based on their established importance for kinase function. Red residue labels (and all-red sidechains) indicate positions acetylated by HopZ1a (S84, T87, T125, T177). The β3 lysine, K76 (labeled in grey), was not mutated but its sidechain is shown for comparison to the salt-bridge-forming lysine (K911) from BRI1 (shown in panel B, below). Note that in this model W58 and W193 would be expected to clash with the non-hydrolyzable ATP analogue AMP-PNP and Mn2+ ions if directly superimposed from the template BRI1 structure (shown in panel B). (B) An identical view of the kinase domain active site showing equivalent positions on the template kinase domain structure of the Arabidopsis brassinosteroid receptor kinase, BRI1 (PDB: 5LPV) [35]. Blue residue labels indicate positions aligned with those that were mutated in ZED1 based on their established importance for kinase function. Grey residue labels indicate positions equivalent to ZED1 sites that are acetylated by HopZ1a, as well as the salt-bridge forming β3 lysine, K911. (Note that Q919 and R922 were poorly resolved in the BRI1 structure, hence only the alpha-carbons of their sidechains are visible.) The ATP analogue AMP-PNP is shown as a ‘ball and stick’ representation, and two Mn2+ ions are shown as purple spheres. Images were prepared with UCSF Chimera [85]. (TIF) [file ppat.1007900.s007.tif]

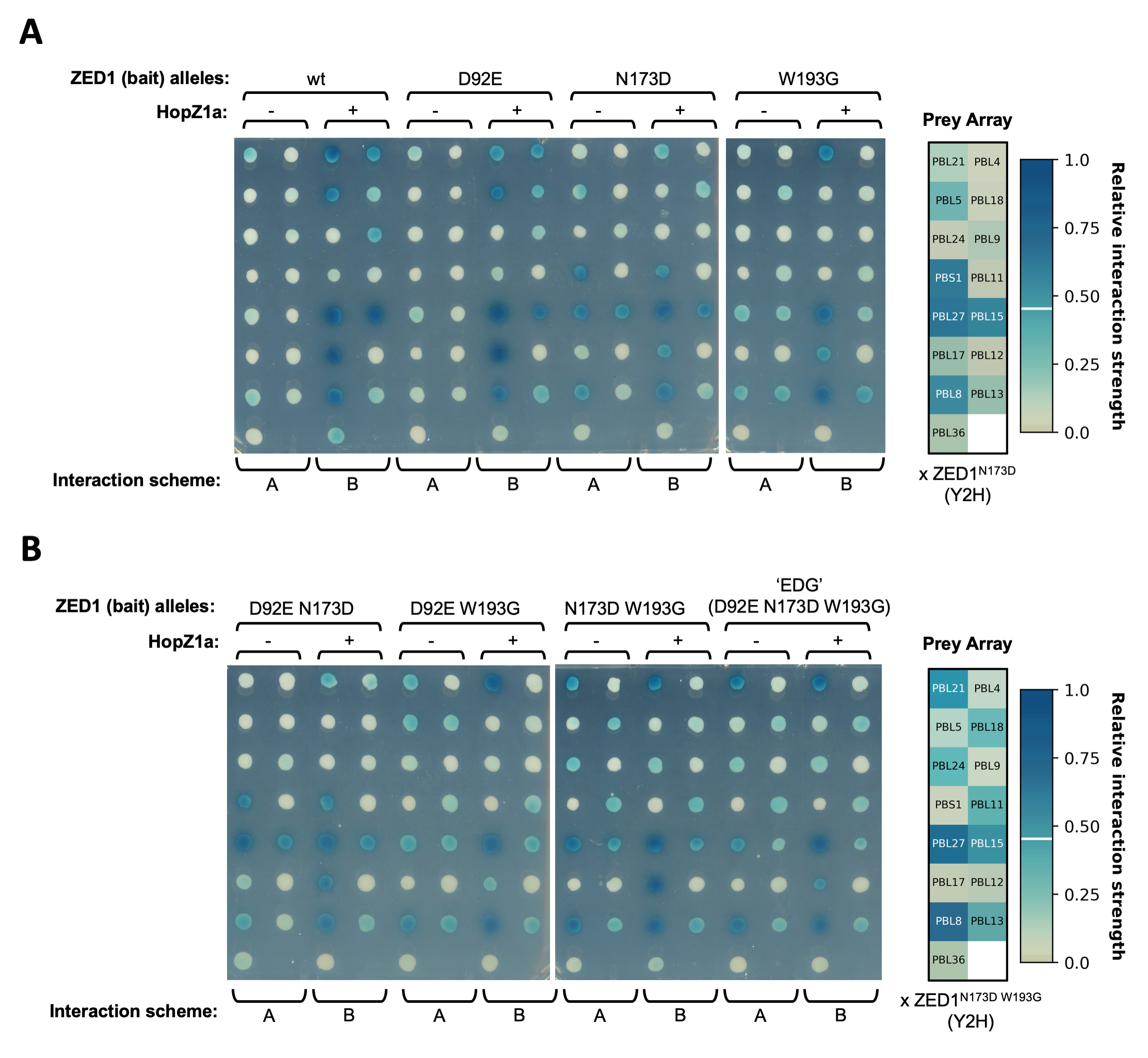

Supplement: S8 Fig — (A) Protein interaction assays to assess the binding affinities of wild-type ZED1 and ZED1 single mutants (sites ‘b’, ‘c’, and ‘d’ in Fig 3) for 15 PBLs in the absence (Y2H; S1 Fig, panel A) or presence (Y3H; S1 Fig, panel B) of HopZ1a. The prey array layout at right indicates the relative strength of PBL interactions with ZED1N173D in the absence of effector. The colours of array labels at each position (black or white) are determined by the relative interaction strength, as described in S2 Fig. (B) Double and triple mutant ZED1 alleles combining the mutations shown in panel A (sites ‘bc’, ‘bd’, ‘cd’, and ‘bcd’ in Fig 3) were likewise tested against the same array of 15 PBLs in both Y2H and Y3H contexts. The prey array layout shown at right indicates the relative strength of PBL interactions with ZED1N173D W193G in the absence of effector. Interaction schemes refer to S1 Fig. (TIF) [file ppat.1007900.s008.tif]

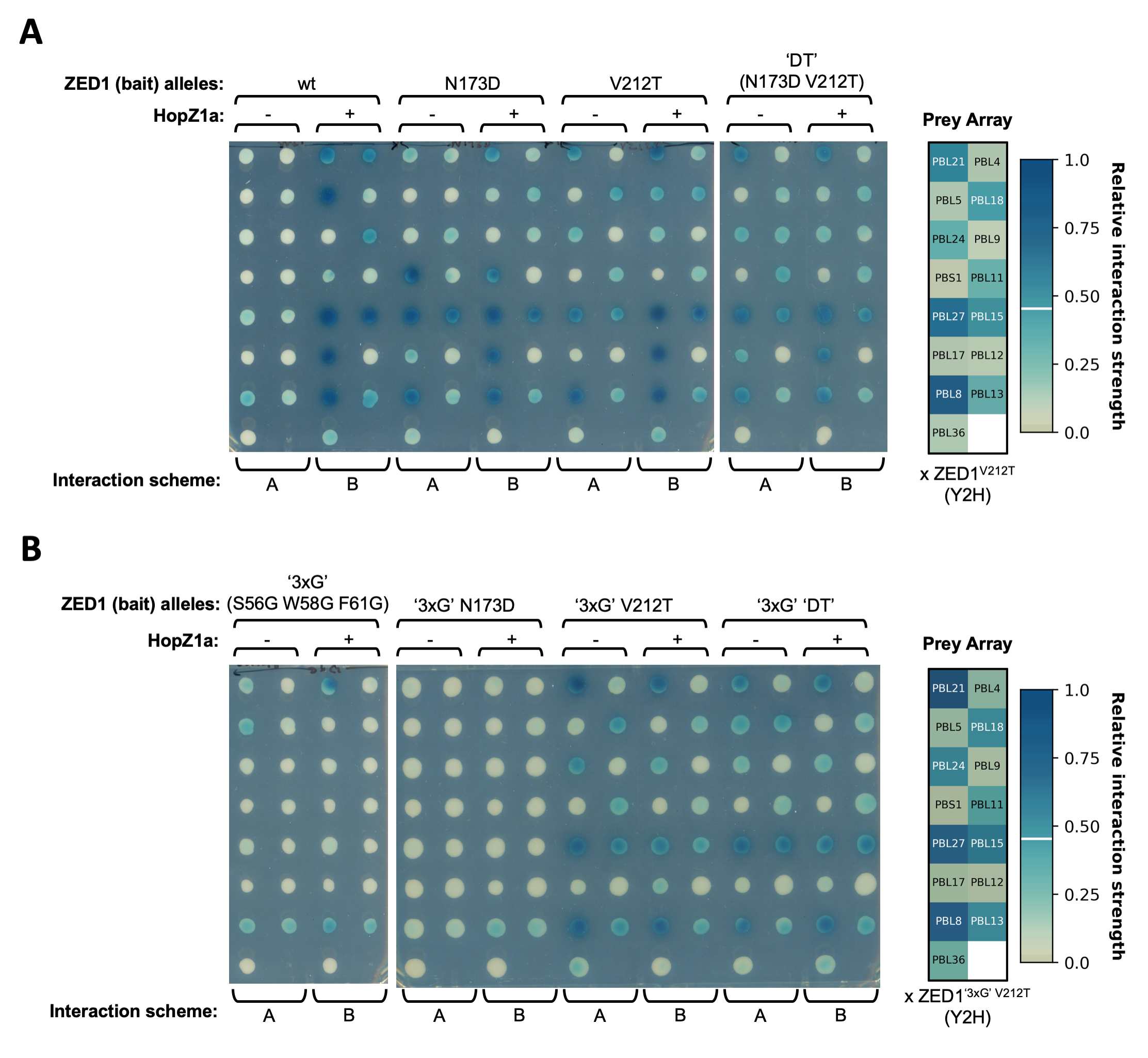

Supplement: S9 Fig — (A) Protein interaction assays to assess the binding affinities of ZED1wt, single mutants (ZED1N173D, ZED1V212T; sites ‘c’, ‘e’ in Fig 3), and a double mutant, (ZED1“DT”; sites ‘ce’ in Fig 3) for 15 PBLs in Y2H and Y3H contexts. The prey array layout shown at right indicates the relative strength of PBL interactions with ZED1V212T in the absence of effector. The colours of array labels at each position (black or white) is determined by the relative interaction strength, as described in S2 Fig. (B) A ZED1 triple mutant (“3xG”; S56G W58G F61G) was tested against the same 15 PBLs, either alone or combined with the single and double mutants shown in panel A. The prey array layout shown at right indicates the relative strength of PBL interactions with ZED1‘3xG’ V212T in the absence of effector. Interaction schemes refer to S1 Fig. (TIF) [file ppat.1007900.s009.tif]

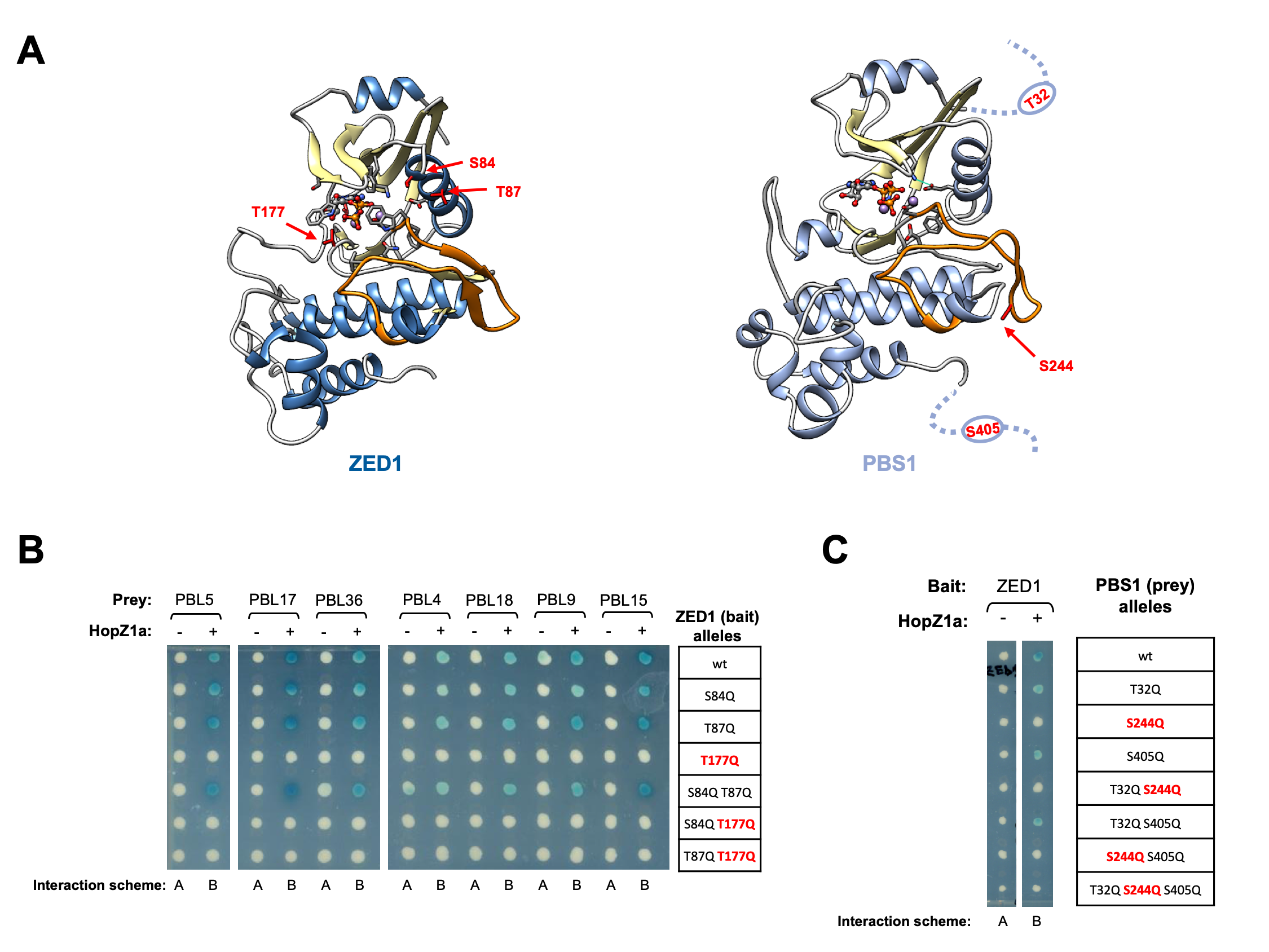

Supplement: S10 Fig — (A) Structural models of ZED1 and PBS1 showing the positions of HopZ1a-acetylated residues as determined by LC-MS/MS. Note that acetylated PBS1 residues T32 and S405 do not form part of the conserved kinase domain. Images were prepared with UCSF Chimera [85]. (B) Y2H and Y3H assays for protein interactions between seven representative PBL preys and the indicated ZED1 bait alleles (wild-type, and mutants with glutamine substitutions at acetylation sites). (C) Y2H and Y3H assays for protein-protein interactions between wild-type ZED1 bait and the indicated PBS1 prey alleles (wild-type, and mutants with glutamine substitutions at acetylation sites). Interaction schemes refer to S1 Fig. (TIF) [file ppat.1007900.s010.tif]

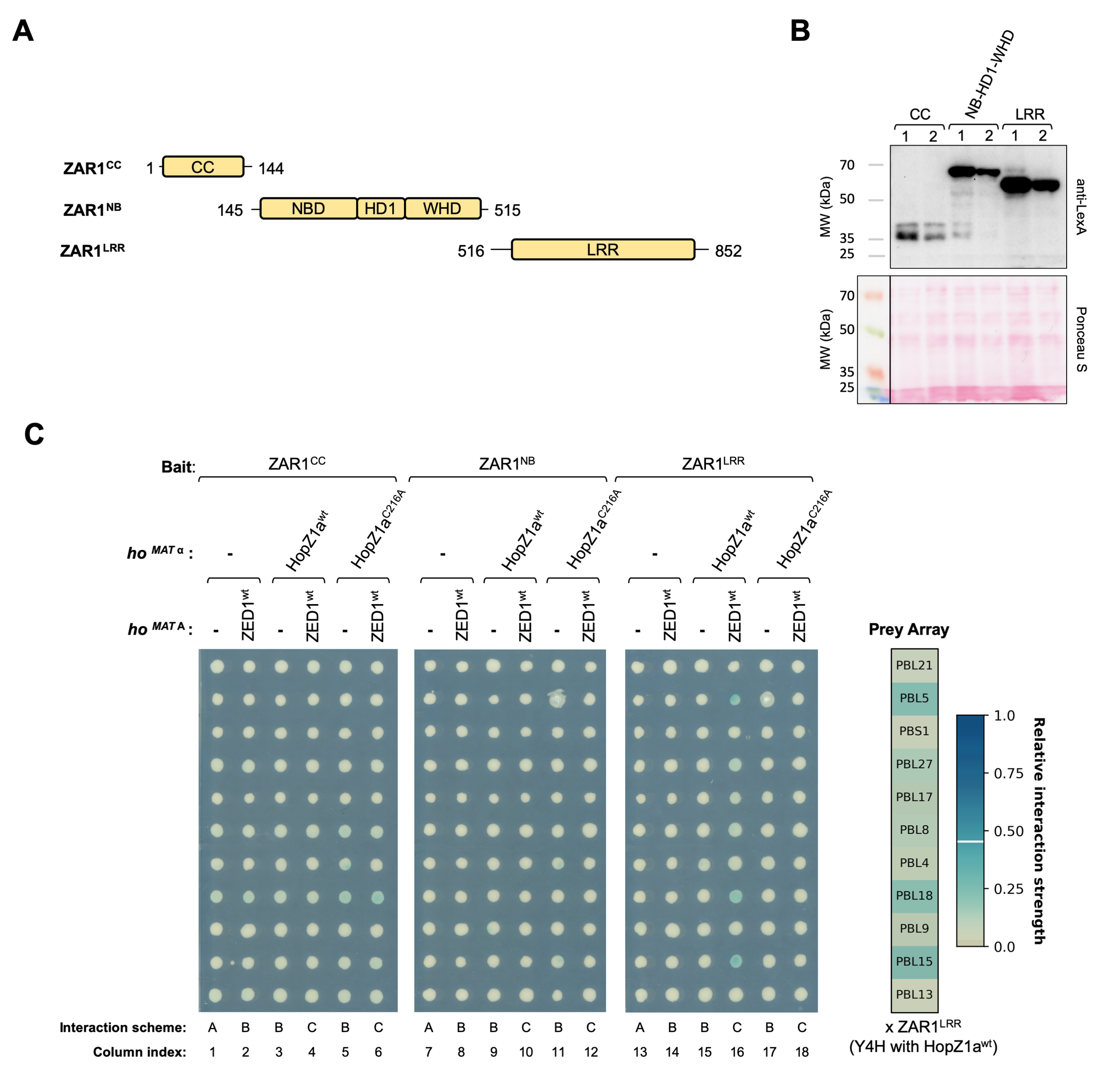

Supplement: S11 Fig — (A) Schematic showing ZAR1 domain truncation boundaries. Subdomains of the central nucleotide-binding region are labeled as described by Wang et al [19,20]: NBD, nucleotide-binding domain; HD1, helical domain 1; and WHD, winged helix domain. (B) Confirmation of expression for each of the three ZAR1 constructs shown in panel A. A western blot of yeast cell lysates probed with serum raised against the LexA DNA-binding domain (top) is compared with Ponceau S staining of the total protein in each lane (bottom). (C) Interactions between ZAR1 bait constructs and 11 PBL preys were assessed in the absence and presence of HopZ1a and/or ZED1 alleles integrated at the ho locus of strain EGY48 (MAT α) or RFY206 (MAT A), corresponding to Y2H (interaction scheme A), Y3H (interaction scheme B), and Y4H (interaction scheme C) assays (S1 Fig). The prey array layout shown at right indicates the relative strength of PBL interactions with ZAR1LRR in the presence of both ZED1 and HopZ1awt. All array labels are black since the relative interaction strengths are all below the threshold described in S2 Fig. (TIF) [file ppat.1007900.s011.tif]

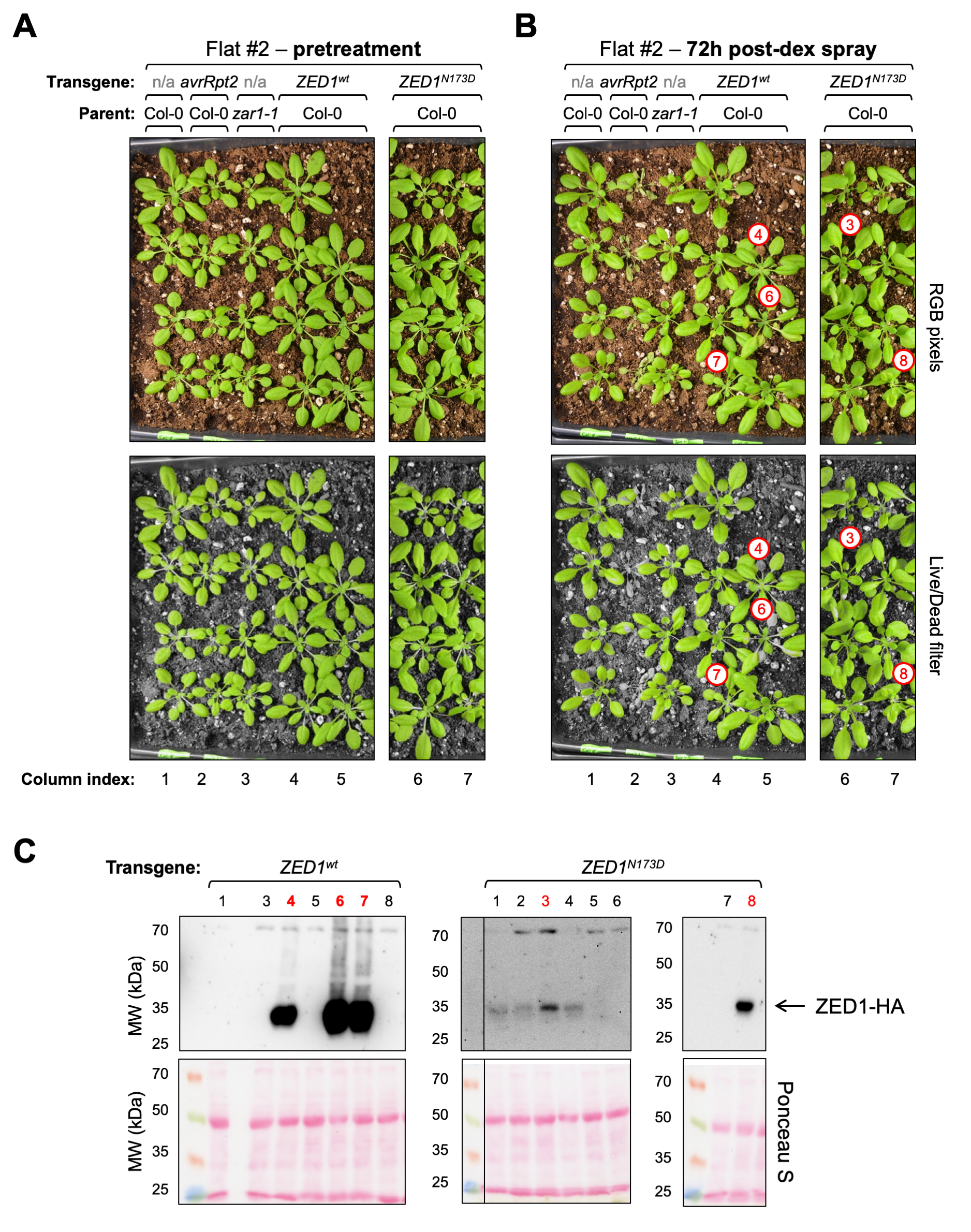

Supplement: S12 Fig — Control plants and BASTA-resistant Arabidopsis transformants bearing dexamethasone-inducible ZED1 alleles (ZED1wt and ZED1N173D) were photographed before treatment (A) and 72 h post-dexamethasone spray (B) and screened for transgene expression (C), as described for ZED1wt and ZED1’DT’ in Fig 6. (TIF) [file ppat.1007900.s012.tif]

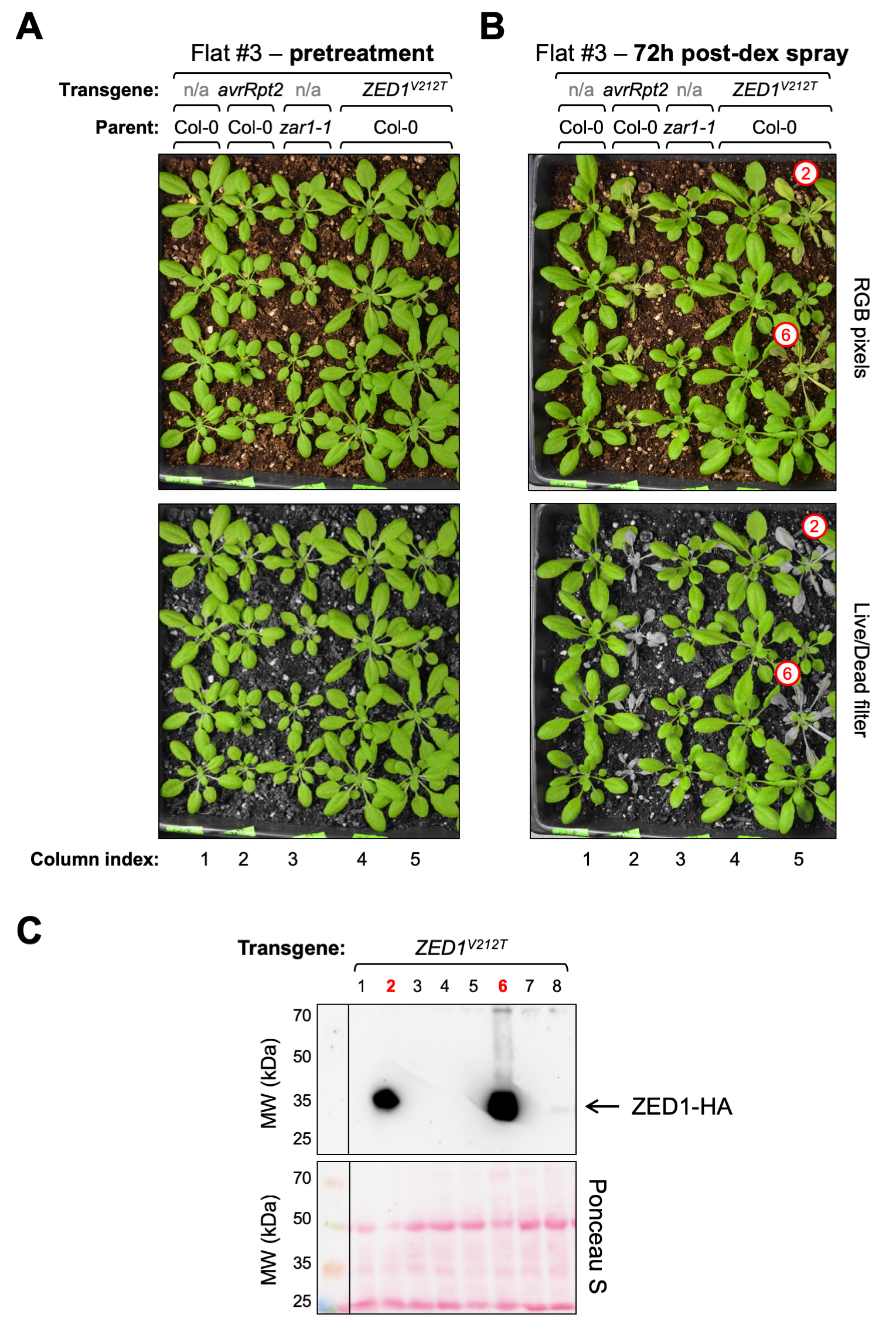

Supplement: S13 Fig — Control plants and BASTA-resistant Arabidopsis transformants bearing dexamethasone-inducible ZED1V212T were photographed before treatment (A) and 72 h post-dexamethasone spray (B) and screened for transgene expression (C), as described for ZED1wt and ZED1‘DT’ in Fig 6. (TIF) [file ppat.1007900.s013.tif]

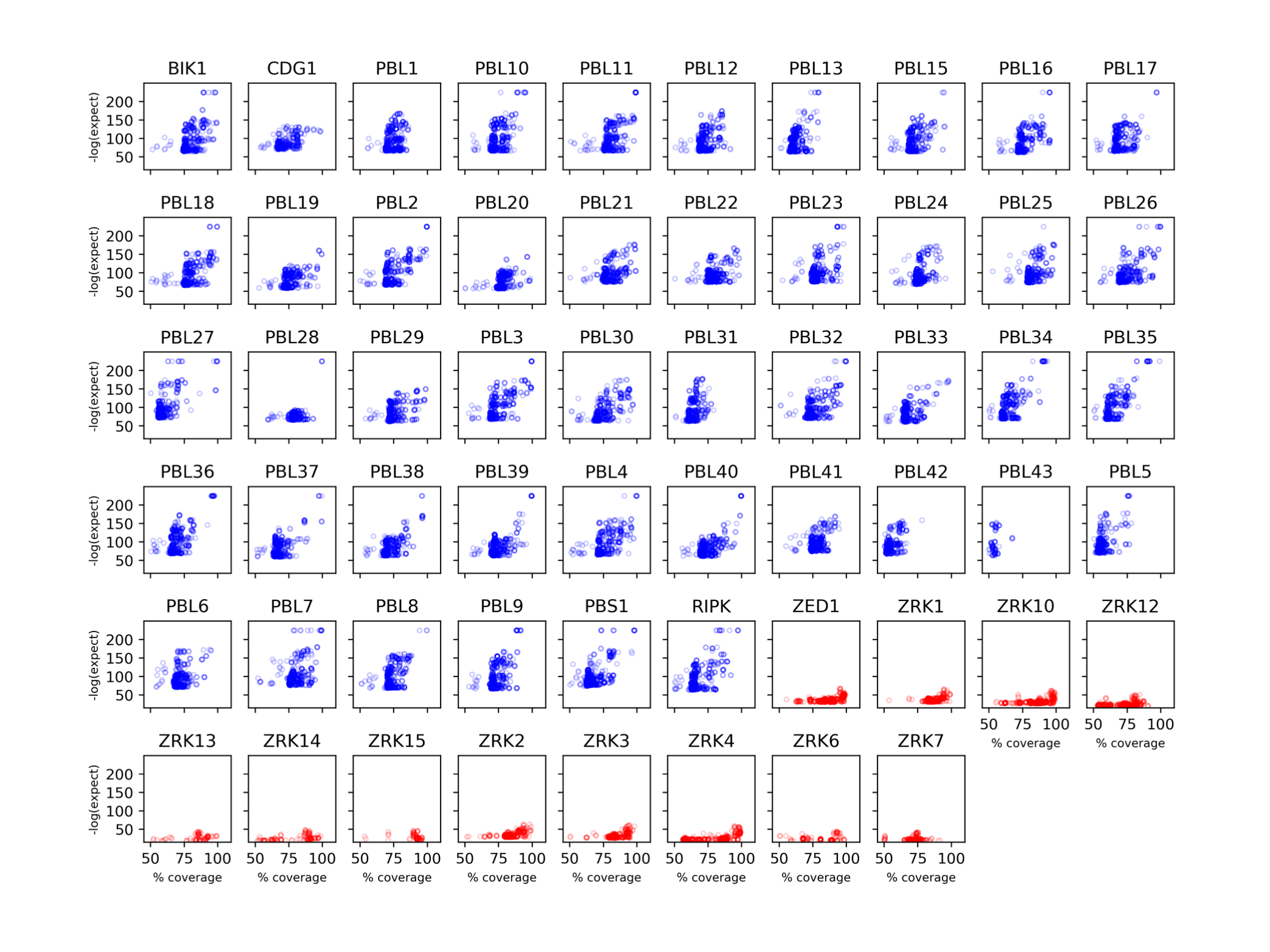

Supplement: S14 Fig — Plotted points represent sequences from Nicotiana spp. (N. tabacum, N. attenuata, N. sylvestris, N. tomentosiformis) with regions homologous to the indicated Arabidopsis query sequences– 46 PBLs (blue) and 12 ZRKs (red). Up to 500 hits for each query are shown where the homologous region spans at least 50% of the length of the query sequence and has an expectation score (E-value) of less than 10e-20. Hits with E-values of zero were arbitrarily assigned values of 10e-225 prior to logarithmic transformation and plotting since the logarithm of zero is not defined. (TIF) [file ppat.1007900.s014.tif]

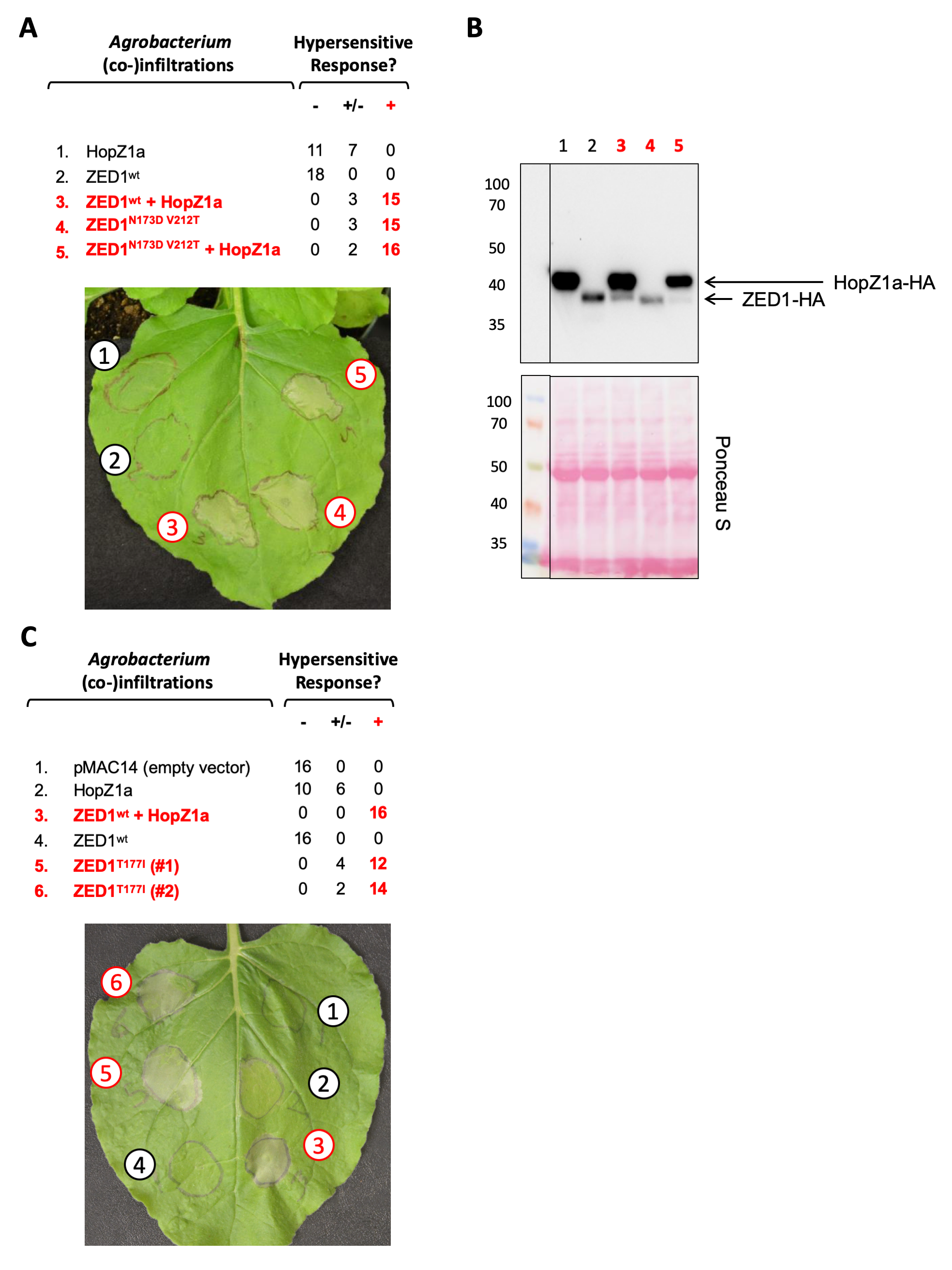

Supplement: S15 Fig — (A) The outcomes of hypersensitive response assays for plants receiving the indicated transformations are indicated as the number of strong (+), weak/partial (+/-), or absent (-) responses observed (top). An N. benthamiana leaf infiltrated with Agrobacterium strains delivering the indicated (combinations of) transgenes, 26 h post-induction of expression by spray with dexamethasone (bottom). The image is representative of three independent experiments wherein six different leaves were infiltrated in the same way. (B) Protein lysates from leaf cores punched from an additional leaf, similarly infiltrated with the same Agrobacterium cell suspensions shown in panel A, were assessed for expression of HA-tagged transgenes. (C) Hypersensitive response outcomes (top), and a representative N. benthamiana leaf infiltrated with Agrobacterium strains delivering the indicated (combinations of) transgenes (bottom), post dexamethasone spray, as for panel A. The image is representative of sixteen different leaves infiltrated in the same way (three independent experiments). (TIF) [file ppat.1007900.s015.tif]

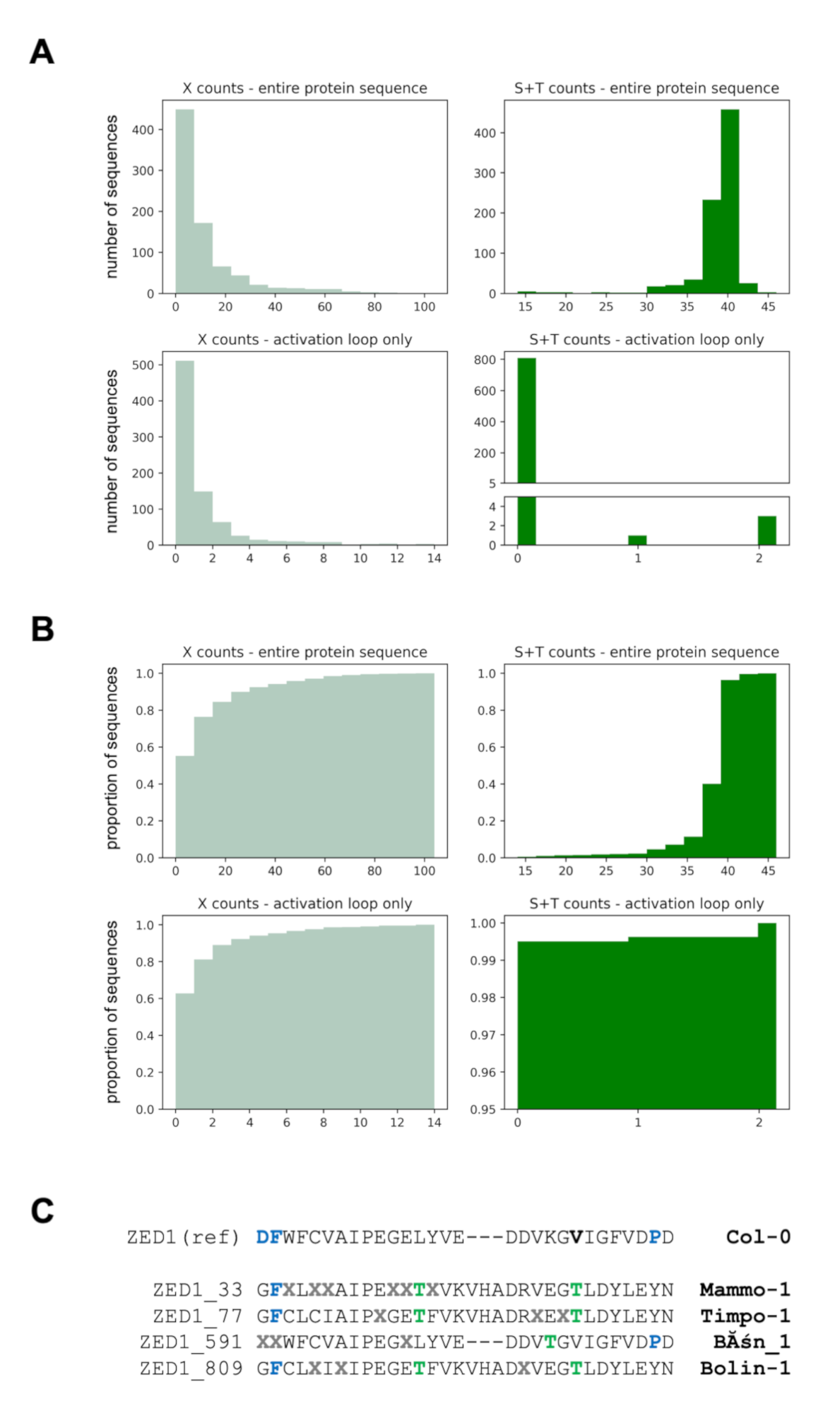

Supplement: S16 Fig — (A) Histograms showing the incidence of ambiguous (left) or potential phospho/acetyl-accepting residues (right) in full-length ZED1 sequences (top) or in activation loop sequences only (bottom). (B) The same data shown in panel A are plotted as cumulative proportions of the total sample size. (C) Aligned ZED1 activation loop sequences for the Col-0 reference ecotype and the four Arabidopsis ecotypes with apparent phospho/acetyl-accepting residues. Matches to canonical DFG and APE motifs defining the activation loop are highlighted in blue, potential phospho/acetyl-accepting residues are highlighted in green, and V212 from the reference Col-0 ecotype is highlighted in bold, black font, as in S6 Fig, panel B. Ambiguous activation loop positions translated as ‘X’ are shown in bold, grey font. (TIF) [file ppat.1007900.s016.tif]

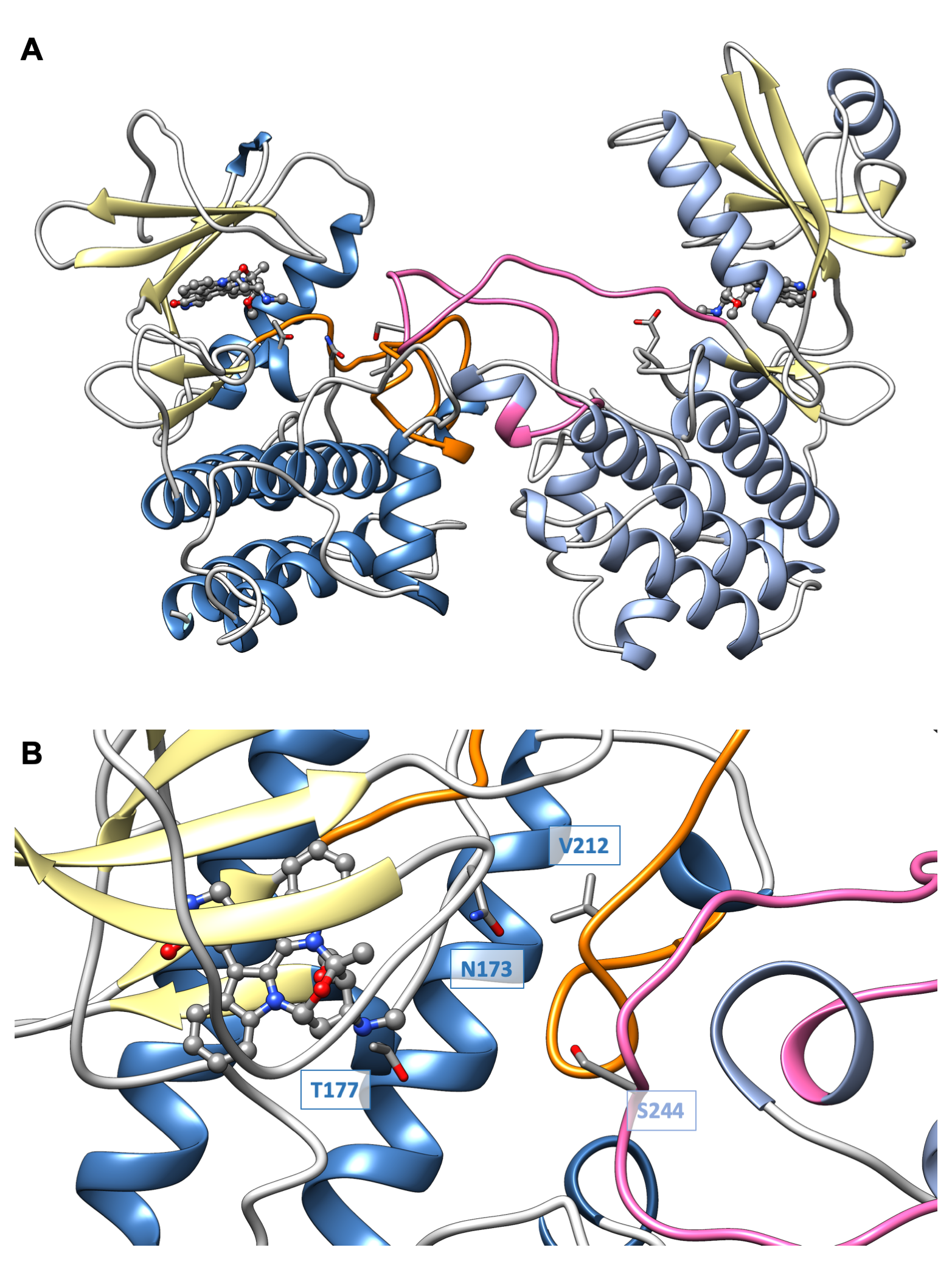

Supplement: S17 Fig — (A) Molecular models of ZED1 (dark blue helices, orange activation loop) and PBS1 (light blue helices, pink activation loop) are superimposed over the asymmetric dimer of inactivated IRAK4 described by Ferrao et al. (PDB: 4U97) [44]. Note that the template IRAK4 structure is hidden except for the co-crystalized inhibitor, staurosporine, which competes with ATP for binding to kinase active sites. (B) A zoomed in view of the same hypothetical ZED1-PBS1 dimer shown in panel A, rotated by 90 degrees to highlight a ‘top-down’ view of the predicted interface between the ZED1 active site and the PBL activation loop acetylation site (S244 in PBS1). Images were prepared with UCSF Chimera [85]. (TIF) [file ppat.1007900.s017.tif]

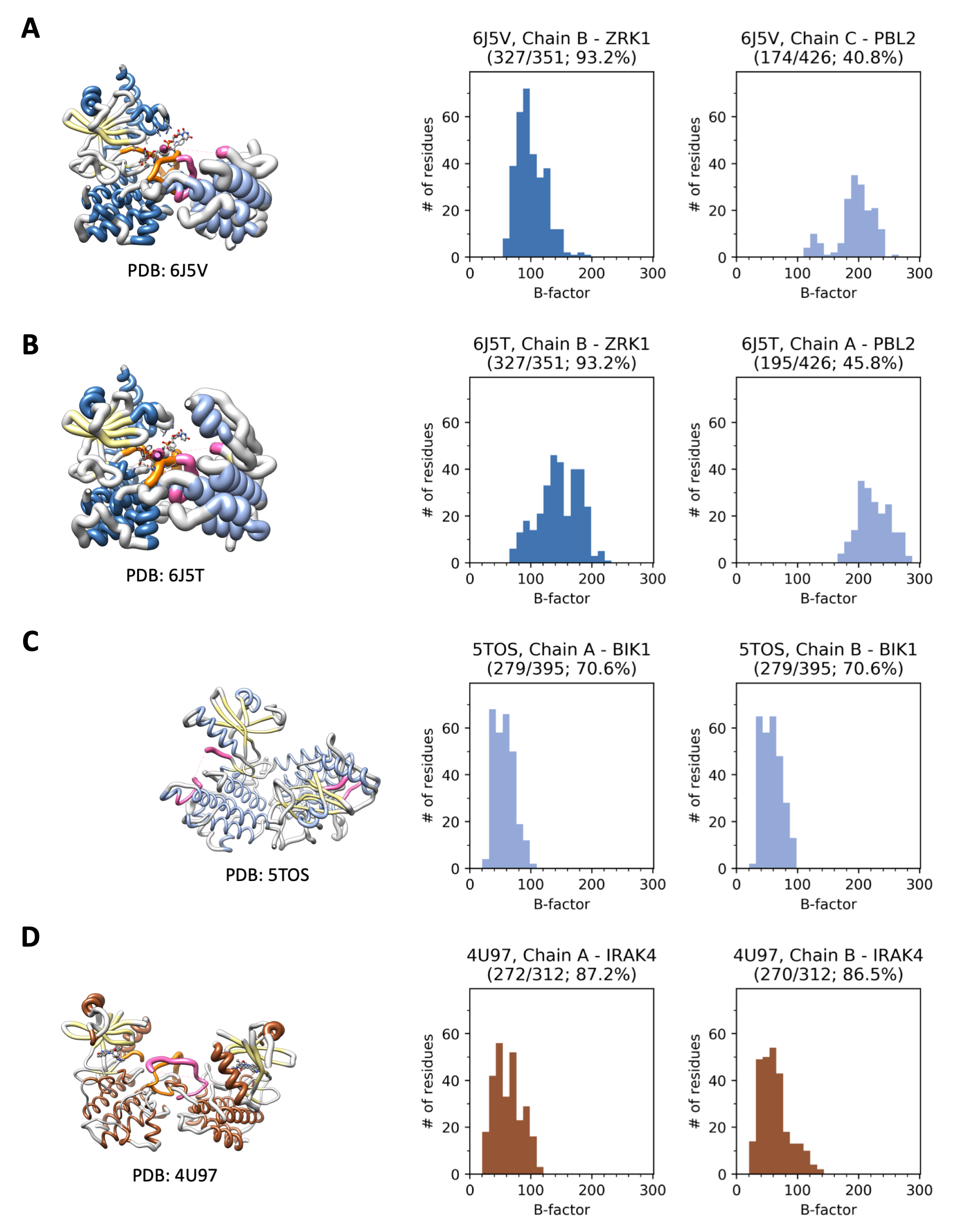

Supplement: S18 Fig — Experimentally-determined structures of kinase hetero- and homo-dimers (left column), and histograms of the average, per-residue B-factors for the two (pseudo)kinase protomers in each structure (middle and right columns, respectively). Kinase domains are depicted as ‘worm’ representations of the polypeptide backbone, with worm diameters proportional to the average B-factor at each position. As for S7 Fig, S10 Fig and S17 Fig, beta-strand elements are coloured yellow, while alpha-helices are coloured according to protein identity: dark blue (ZRK1/RKS1, panels A, B); light blue (PBL2, panels A, B; BIK1, panel C); or brown (IRAK4, panel D). Activation loop sequences are coloured orange or pink. Uridylated side chains of PBL2 are shown in ‘ball-and-stick’ representation. Missing segments not observed in the structures are indicated with dashed lines. The proportion of the total sequence represented by a given structure is indicated above each histogram, expressed both as a fraction and as a percent. (A) The ZRK1/RKS1-PBL2UMP heterodimer from the monomeric nucleotide-free ZAR1-ZRK1/RKS1-PBL2UMP complex (PDB: 6J5V) [19]. (B) The ZRK1/RKS1-PBL2UMP heterodimer from the pentameric ZAR1-ZRK1/RKS1-PBL2UMP resistosome (PDB: 6J5T) [20]. (C) A homodimer of the Arabidopsis PBL kinase, BIK1 (PDB: 5TOS) [55]. (D) A homodimer of a catalytic site mutant (D311N) of the human kinase, IRAK4 (PDB: 4U97) [44]. (TIF) [file ppat.1007900.s018.tif]

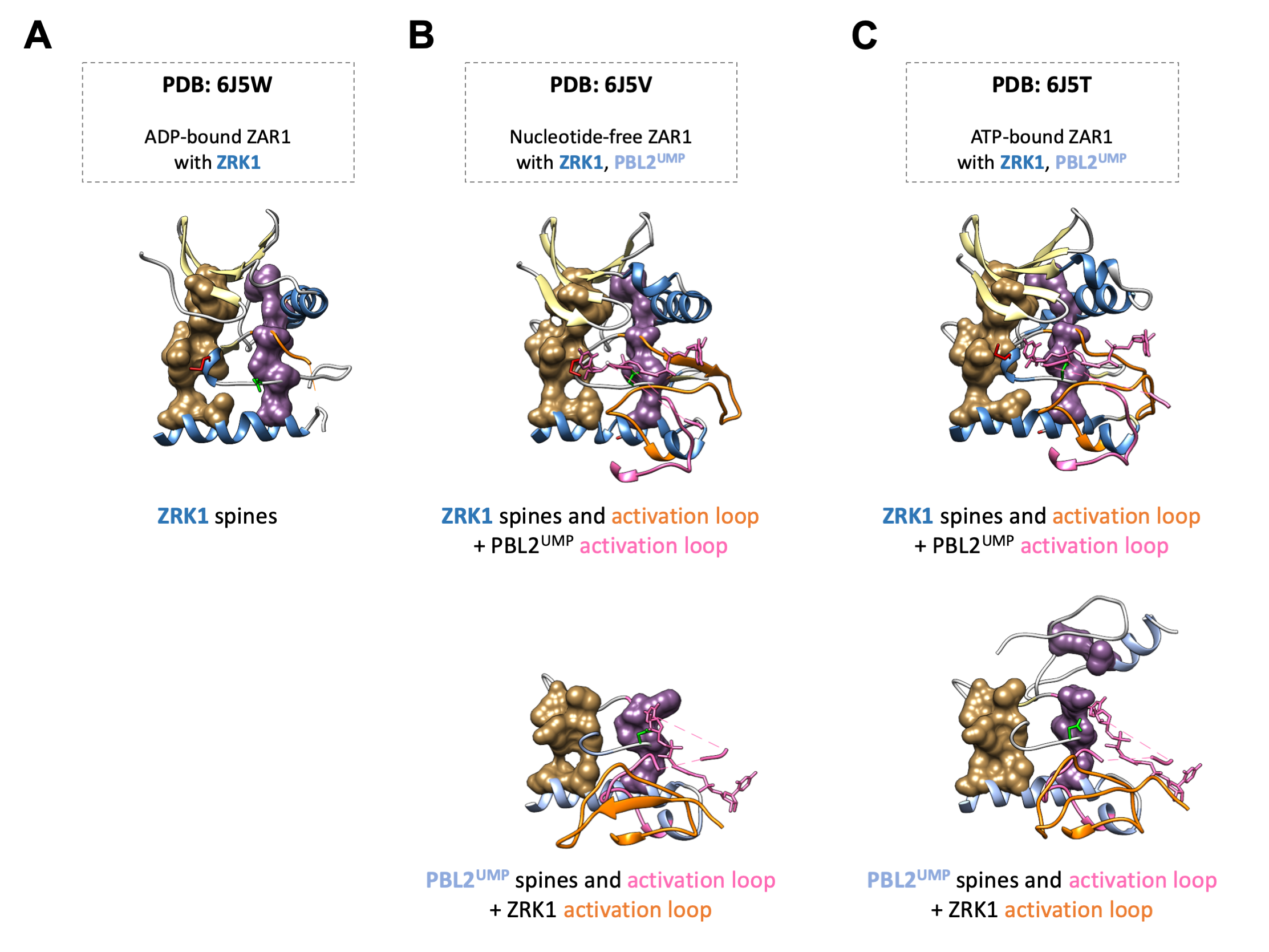

Supplement: S19 Fig — ‘Catalytic’ and ‘regulatory’ spines (‘C-spine’, ‘R-spine’) for ZRK1/RKS1 and PBL2UMP are represented as molecular surfaces in brown (C-spines) and purple (R-spines). For clarity, ribbon representation of the protein sequences is restricted to three regions (where present in the structures): (1) β1-β2-β3-αC-β4-β5, (2) catalytic segment-activation loop, and (3) helix αF, which anchors both the catalytic and regulatory spines. The sidechain of ZRK1/RKS1 residue M195 (equivalent to ZED1 acetylation site T177) is shown in red. The catalytic aspartate residues for ZRK1/RKS1 (D191) and PBL2 (D219) are shown in green. Missing (unstructured) segments are indicated with dashed lines. C-spine and R-spine residues for ZRK1/RKS1 and PBL2 are presented in S6 Table along with the corresponding positions from ZED1 and PKA [91]. Note that ZAR1 is also present in each of these structures (panels A-C) but is not shown for clarity. (A) ZRK1/RKS1 in complex with ADP-bound ZAR1—PDB entry 6J5W (B) ZRK1/RKS1 (top) and PBL2UMP (bottom) from a complex with nucleotide-free ZAR1—PDB entry 6J5V. (C) ZRK1/RKS1 (top) and PBL2UMP (bottom) from a complex with ATP-bound ZAR1—PDB entry 6J5T. (TIF) [file ppat.1007900.s019.tif]

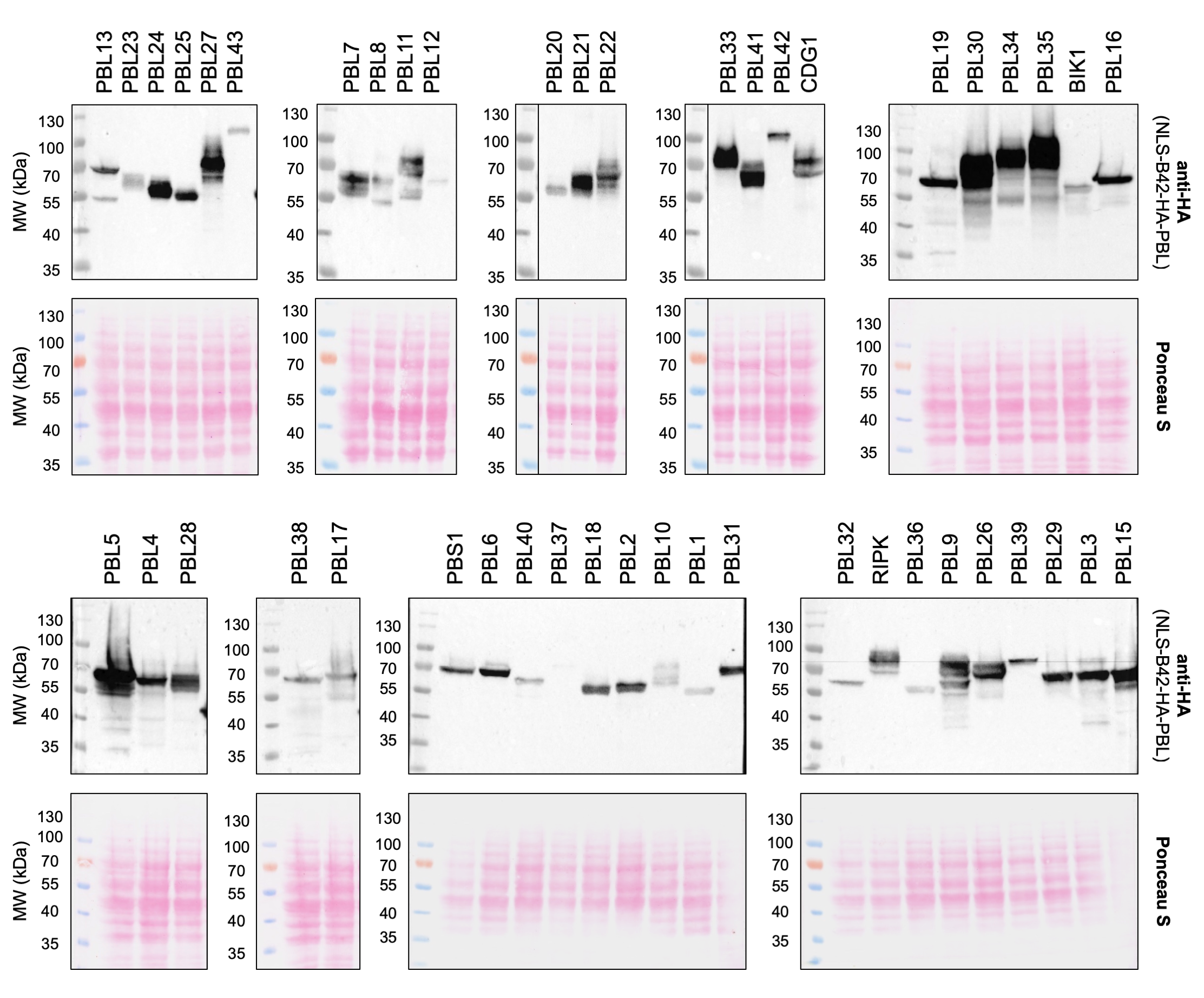

Supplement: S20 Fig — Western blots of yeast cell lysates expressing the indicated prey-PBL fusion proteins (top). Ponceau S-stained nitrocellulose membranes showing the total protein loaded in each lane (bottom). (TIF) [file ppat.1007900.s020.tif]

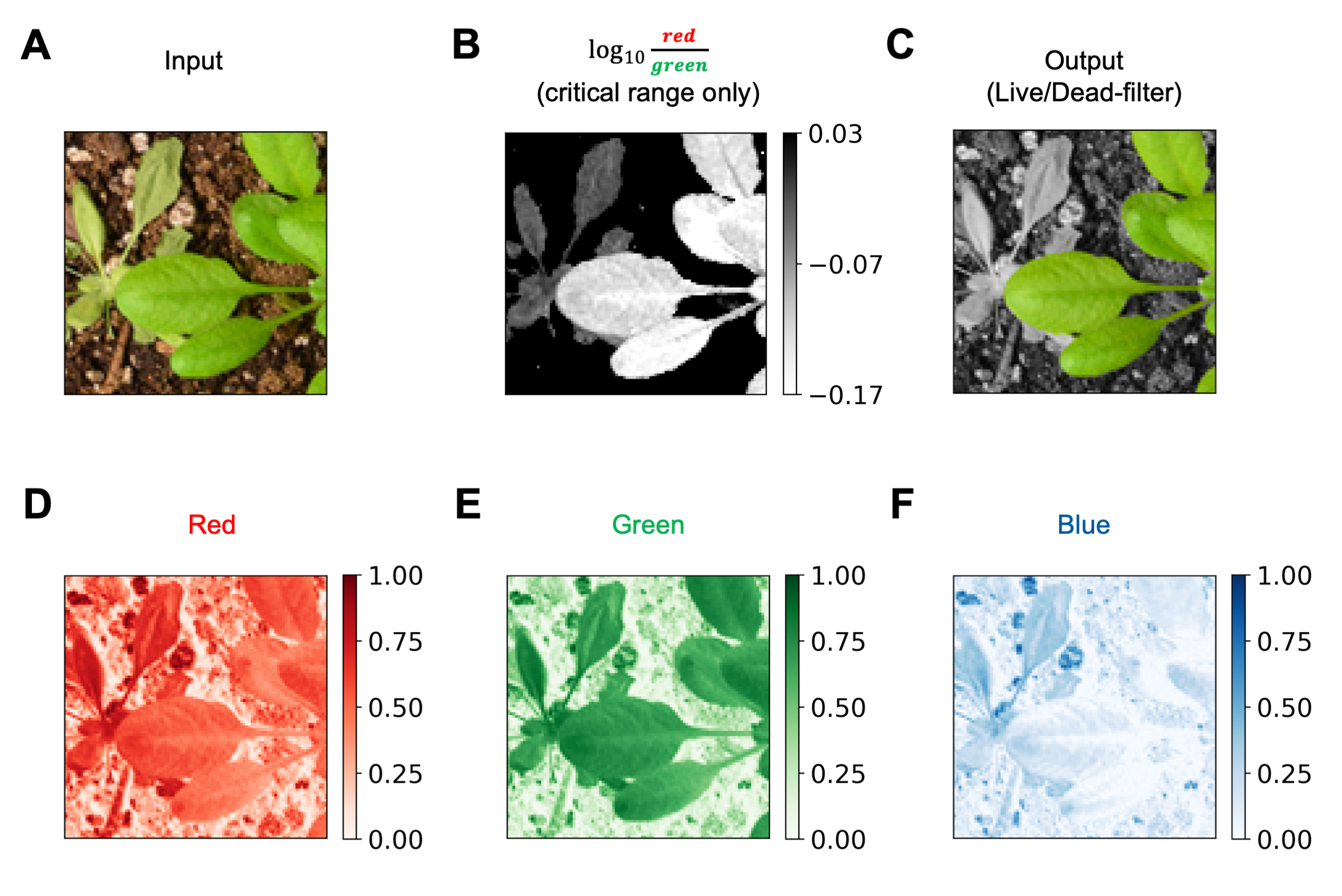

Supplement: S21 Fig — Pixels from an input image (A) are subjected to threshold-masking of full-colour RGB pixels (C) based on the ratio between red and green channel intensities (B). False-colour representations of the absolute pixel intensities for individual red (D), green (E), and blue (F) channels are shown below. (TIF) [file ppat.1007900.s021.tif]
